# Dynamic susceptibility contrast perfusion-weighted and diffusion-weighted magnetic resonance imaging findings in pilocytic astrocytoma and H3.3 and H3.1 variant diffuse midline glioma, H3K27-altered

**DOI:** 10.1371/journal.pone.0288412

**Published:** 2023-07-14

**Authors:** Ryo Kurokawa, Mariko Kurokawa, Akira Baba, John Kim, Ashok Srinivasan, Toshio Moritani

**Affiliations:** Division of Neuroradiology, Department of Radiology, University of Michigan, Ann Arbor, MI, United States of America; Goethe University Hospital Frankfurt, GERMANY

## Abstract

**Objective:**

This study compared the dynamic susceptibility contrast (DSC) magnetic resonance imaging parameters and apparent diffusion coefficient (ADC) between pilocytic astrocytoma (PA) and diffuse midline glioma, H3K27-altered (DMG) variants.

**Methods:**

The normalized relative cerebral blood volume (nrCBV), normalized relative flow (nrCBF), percentile signal recovery (PSR), and normalized mean ADC (nADCmean) of 23 patients with midline PAs (median age, 13 years [range, 1–71 years]; 13 female patients) and 40 patients with DMG (8.5 years [1–35 years]; 19 female patients), including 35 patients with H3.3- and five patients with H3.1-mutant tumors, treated between January 2016 and May 2022 were statistically compared.

**Results:**

DMG had a significantly lower nADCmean (median: 1.48 vs. 1.96; *p* = 0.00075) and lower PSR (0.97 vs. 1.23, *p* = 0.13) but higher nrCBV and nrCBF (1.66 vs. 1.17, *p* = 0.058, respectively, and 1.87 vs. 1.19, *p* = 0.028, respectively) than PA. The H3.3 variant had a lower nADCmean than the H3.1 variant (1.46 vs. 1.80, *p* = 0.10).

**Conclusion:**

DMG had lower ADC and PSR and higher rCBV and rCBF than PA. The H3.3 variant had a lower ADC than the H3.1 variant. Recognizing the differences and similarities in the DSC parameters and ADC between these tumors may help presurgical diagnosis.

## Introduction

Pilocytic astrocytoma (PA) and diffuse midline glioma, H3K27-altered (DMG) are primary brain tumors that develop most frequently in children. PA is a potentially curable and less vascularized central nervous system (CNS) World Health Organization (WHO) grade 1 tumor [1]. PA is histologically characterized by a biphasic pattern consisting of densely populated components of differentiated pilocytes with bipolar processes and various degrees of loosely textured components with abundant myxoid stroma [2]. PA is molecularly characterized by frequent fusions between *KIAA1549* and *BRAF* [2]. PA frequently occurs in midline structures, such as the optic nerve, hypothalamus, thalamus, cerebellar vermis, and brainstem, although it also develops in the cerebellar hemispheres [3]. In contrast, DMG is a CNS WHO grade 4 tumor with a dismal survival prognosis characterized by a midline location and histone H3 gene alterations [1]. Approximately 70% of DMGs harbor a mutated *H3F3A* gene that encodes the H3.3 variant, whereas most of the remaining DMGs have mutated *HIST1H3B* or *HIST1H3C* genes, which encode the H3.1 variant [4]. The survival prognosis differs between the variants. The median overall survival of the H3.3 variant is 9 months, whereas it is 15 months in the H3.1 variant [5]. The prognosis of the natural course and management required in PA and DMG are substantially different; however, these tumor types tend to occur in the similar regions of the pediatric population, and the imaging findings may overlap (especially between predominantly solid PA [6] and DMG), occasionally rendering the diagnosis challenging. Furthermore, due to the tumor localization in the vital midline structures, even the most minimally invasive stereotactic needle biopsy puts the patients at risk of complications, including neurologic deficit, intraparenchymal hemorrhage, and death [7]. Although all the included cases in the present study were pathologically diagnosed, the midline-located pediatric tumors are often not biopsied and therefore managed without pathological evidence [8]. Hence, it would be clinically beneficial to improve the accuracy of preoperative imaging diagnosis of the midline-located tumors including PA and DMG.

DSC perfusion MRI (DSC-MRI) and diffusion-weighted imaging (DWI) play an important role in brain tumor typing and grading. With regard to PA, Zhou et al. [9] (supratentorial PA vs. extraventricular ependymoma), Kurokawa et al. [10] (infratentorial PA vs. medulloblastoma), and Ho et al. [11] (PA vs. pilomyxoid astrocytoma) reported the effectiveness of a combined approach using DSC parameters and ADCs derived from DWI to differentiate between the tumors. However, limited reports exist regarding the diagnostic performance of these sequences, especially DSC-MRI, in differentiating DMG from other tumor types, including PA.

Furthermore, little is known about the characteristics and differences of the DSC parameters and ADCs in the H3.3 and H3.1 variants of DMG, although their prognoses differ. Leach et al. [12] compared the presence of diffusion restriction between 28 H3.3 DMGs and 11 H3.1 DMGs and reported diffusion restriction in 18/28 (64.3%) cases and 6/11 (54.5%) cases, respectively. However, the definition of diffusion restriction was not determined numerically in their study.

With the recent development of molecularly targeted therapies for DMG and the indication for advanced therapies such as chimeric antigen receptor T cell therapy [13], recognizing differences and similarities in the neuroimaging characteristics of DMG and its mimickers has become more important than ever to ensure appropriate management. In this study, we aimed to evaluate and compare the DSC parameters and ADC values between midline PA and DMG and between the H3.3 and H3.1 variants of DMG.

## Methods

The study was approved with a waiver of the requirement for informed consent owing to the retrospective study design by the ethics committee of the University of Michigan. Data were acquired in compliance with all applicable Health Insurance Portability and Accountability Act regulations, and all methods were performed in accordance with relevant guidelines and regulations. Data were de-identified prior to any analysis.

### Patients

Between January 2016 and May 2022, we searched the electronic database of our hospital. The inclusion and exclusion criteria were as follows:

Inclusion criteria:

- Pathologically diagnosed tumors
- Pretreatment MRI with DSC-MRI or DWI data available
- The tumor was in the midline regions of the brain
- For DMGs, H3.3 or H3.1 variants were diagnosed via gene sequencing

Exclusion criteria:

- Extracranial tumors
- Patients with neurofibromatosis type 1 (in order to remove bias during imaging analysis and to reduce heterogeneity in the patient population)
- PA with H3K27 mutation
- DMG with an unknown H3.3 or H3.1 status
- H3-wildtype DMG with EZH inhibitory protein overexpression [14].

### MRI scanning protocol

For the MRI examinations of the brain, 1.5T and 3T MRI systems (Ingenia 1.5T and Ingenia 3T, Achieva: Philips Healthcare, Eindhoven, the Netherlands; Signa HDxt, MAGNETOM Vida: Siemens, Erlangen, Germany) were performed with the patient in supine position. For DSC-MRI, a power injector was used to administer an intravenous bolus of 20 mL of gadobenate dimeglumine (Multihance; Bracco Diagnostics, Singen, Germany) or gadoteridol (ProHance; Bracco Diagnostics) with a flow rate of 5.0 mL/s through a peripheral vein in the arm, followed by a 20 mL saline flush. An additional 5 mL of the contrast was administered 5 minutes before the dynamic perfusion scan. The dose of the total contrast material was determined to be 0.2 mL/kg (ProHance) for pediatric patients.

The following parameters were used for fast field echo T2*-weighted imaging: plane, axial; repetition time, 1500–1840 ms; echo time, 30–50 ms; number of excitations, 1; slice thickness, 4–5 mm; slice increment, 5–5.2 mm; flip angle, 40°–90°; field of view, 226–235 mm; matrix, 128 × 128–144 × 144; and timepoints, 40–70. The following parameters were used for DWI: b value = 0, 1000 s/mm^2^; repetition time, 3500–5900 ms; echo time, 58.2–91.2 ms; number of excitations, 1 or 2; slice thickness, 4–5 mm; slice increment: 4.4–5 mm; flip angle, 90°–180°; field of view, 227–251 mm; and matrix, 176 × 176–320 × 320.

### Quantitative DSC-MRI analyses

OleaSphere (version 3.0; Olea Medical, La Ciotat, France) was used to conduct the quantitative DSC-MRI analyses. The data were processed with motion artifact correction using rigid-body registration. Cluster analysis techniques were used to automatically calculate the arterial input function. A time-insensitive block-circulant singular-value decomposition [15] was used to perform deconvolution of the arterial input. Voxel-wise division of the area under the concentration-time curve by the area under the arterial input function was used to generate the whole-brain leakage-corrected relative CBV (rCBV) and relative CBF (rCBF) maps. One board-certified radiologist with 9 years of experience in neuroradiology, under the direct supervision of another board-certified radiologist with 13 years of experience in neuroradiology, carefully delineated the regions-of-interest (ROIs) by freehand on every axial slice of the perfusion maps depicting the tumor to generate the volumes-of-interest (VOIs) while excluding cystic, necrotic, or hemorrhagic regions and vessels, by referring simultaneously scanned conventional sequences including post-contrast fat-saturated T1-weighted imaging or by copying ROIs placed on the post-contrast fat-saturated T1-weighted imaging as appropriate. Another 10–15mm diameter circular ROI was placed as the reference over the supratentorial normal-appearing contralateral (in cases with supratentorial tumors) or either side (in cases with infratentorial tumors) of white matter to correct for age- and patient-dependent variations in perfusion parameters [16]. The radiologists were blinded to the tumor pathology. The VOI and reference ROI on the perfusion maps were transposed on the rCBV and rCBF maps. The normalized rCBV (nrCBV) and normalized rCBF (nrCBF) were calculated by dividing the mean rCBV and mean rCBF of the tumor by those of the reference ROIs.

The following formula was used to calculate the percentage signal recovery (PSR) [17]:

$$PSR=100\%\times \frac{\left[S1-Smin\right]}{\left[S0-smin\right]}$$

where *S0* is the baseline signal intensity averaged over the first 10 timepoints, *S1* is the tail averaged over the last 10 timepoints, and *S*min is the minimum T2*-weighted signal intensity in the dynamic series. To calculate the PSR within the slice depicting the largest areas of the tumors, a circular ROI (30–40 mm^2^) was placed within the solid components of the tumors.

### Quantitative ADC analyses

OleaSphere (Olea Medical) was used to generate the ADC maps. VOIs were generated on the solid components of the tumors using the same method that was used for DSC-MRI analysis. The reference ROI was placed in the normal-appearing white matter, and the ADC values were divided by the mean ADC of the reference ROIs to calculate the normalized mean ADC (nADCmean) for each VOI of the tumors.

### Statistics

The Mann–Whitney *U* test and Fisher’s exact test were used to compare the age at diagnosis and sex between PA and DMG and between the H3.3 and H3.1 variants, respectively. The Mann–Whitney *U* test was used to compare the DSC parameters (i.e., nrCBV, nrCBF, and PSR) between PA and DMG and the nADCmean between PA and DMG and between the H3.3 and H3.1 variants. Only one patient with the H3.1 variant underwent DSC-MRI; therefore, the DSC parameters were not compared between the H3.3 and H3.1 variants. Shapiro–Wilk tests were conducted to confirm the normality of the distribution for numerical parameters. The Bonferroni method was used to correct family-wise error. Two-sided *p*-values < 0.05/18 (i.e., 0.0028) were considered statistically significant. R software (version 4.1.1; R Foundation for Statistical Computing, Vienna, Austria) was used for all statistical analyses.

## Results

The demographic and imaging data are summarized in **Table 1**.

**Table 1:** Demographic and imaging data.

	Pilocytic astrocytoma	Diffuse midline glioma, H3K27-altered	H3.3	H3.1	*p*-value (PA vs. DMG)	*p*-value (H3.3 vs. H3.1)	*p*-value (PA vs. H3.3)	*p*-value (PA vs. H3.1)
	n = 23	n = 40	n = 35	n = 5				
Age (median years [range])	13 [1–71]	8.5 [1–35]	11 [1–35]	7 [4–9]	0.74	0.16	0.96	0.23
Sex (M:F)	10:13	21:19	20:15	1:4	0.60	0.17	0.42	0.62
Site								
Brainstem	11	20	16	4				
Thalamus	3	18	17	1				
**Other	9	2	2	0				
DSC-MRI	n = 14	n = 16	n = 15	n = 1				
nrCBV (median [range])	1.17 [0.47–2.85]	1.66 [0.74–4.86]	1.67 [0.74–4.86]	1.76	0.058		0.063	
nrCBF (median [range])	1.19 [0.78–3.00]	1.87 [0.91–11.2]	1.87 [0.91–11.2]	1.56	0.028		0.023	
PSR (median [range])	1.23 [0.46–3.52]	0.97 [0.55–2.78]	1.00 [0.55–2.78]	0.78	0.13		0.172	
DWI	n = 23	n = 40	n = 35	n = 5				
nADCmean (median [range])	1.96 [1.43–3.5]	1.48 [1.11–2.76]	1.46 [1.11–2.76]	1.80 [1.59–2.35]	**0.00075** *	0.10	**0.00020** *	0.68

* Indicates a statistically significant value.

DMG = diffuse midline glioma, H3K27-altered; DSC = dynamic susceptibility contrast; DSC-MRI = dynamic susceptibility contrast magnetic resonance imaging; DWI = diffusion-weighted imaging; nADCmean = normalized mean apparent diffusion coefficient; nrCBF = normalized relative cerebral blood flow; nrCBV = normalized relative cerebral blood volume; PA = pilocytic astrocytoma; PSR = percentage signal recovery.

** Other: cerebellum (n = 9) in PA; septum pellucidum (n = 1) and basal ganglia (n = 1) in DMG (H3.3)

### Demographic data

In total, 23 patients with midline PA (median age, 13 years; range, 1–71 years; 13 female patients) and 40 patients with DMG (median age, 8.5 years; range, 1–35 years; 19 female patients) were included in this study. The DMG group was further divided into 35 H3.3 cases (median age, 11 years; range, 1–35 years; 15 female patients) and five H3.1 cases (median age, 7 years; range, 4–9 years; four female patients). No significant difference was observed in terms of age and sex of the patients between PA and DMG or between the H3.3 and H3.1 variants.

### Imaging findings

**Fig 1:**
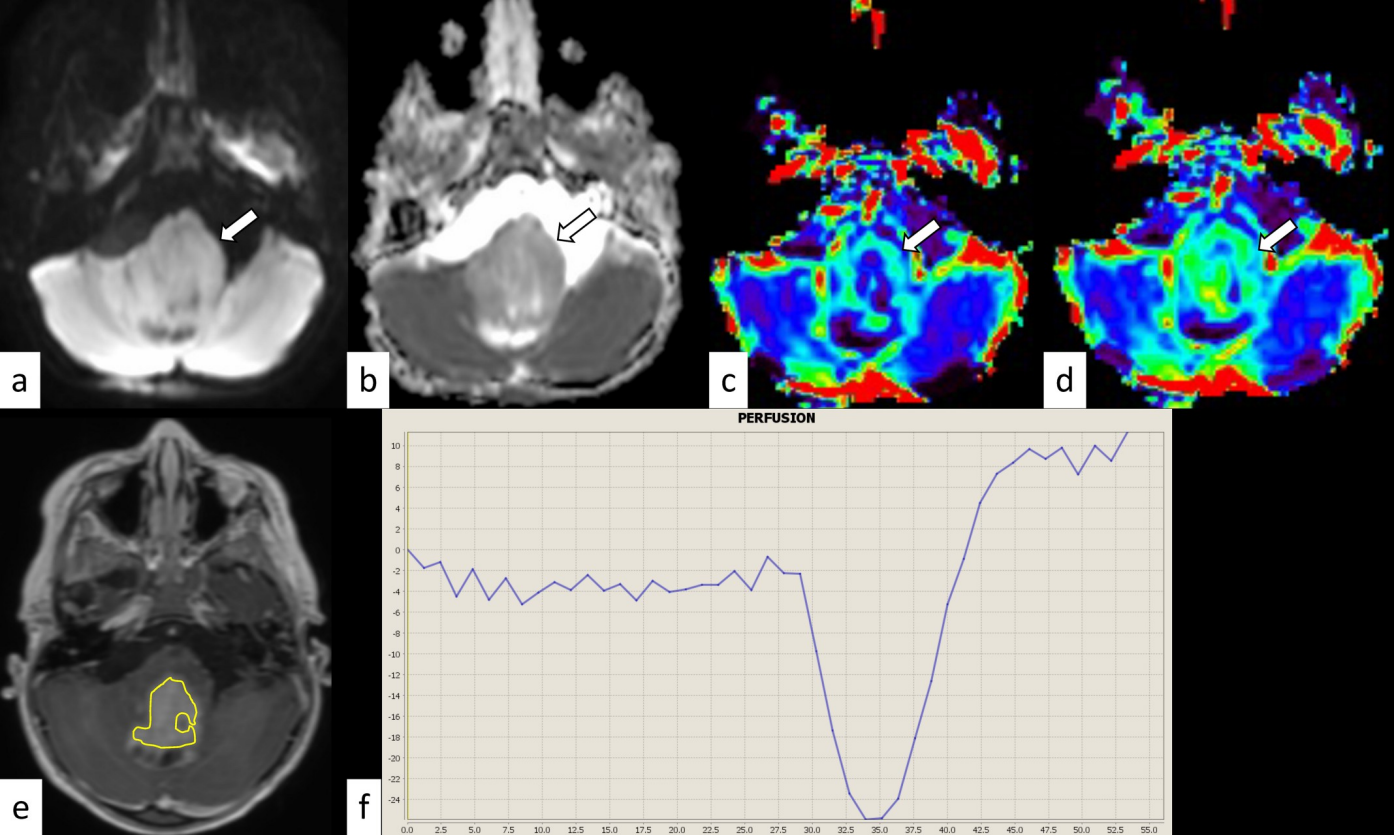
Pontine pilocytic astrocytoma in a 3-year-old boy. The mass shows isointensity on diffusion-weighted imaging (a, arrow) and an increased apparent diffusion coefficient (nADCmean = 1.52) (b, arrow). The normalized relative cerebral blood volume and flow are 1.79 and 1.90, respectively (c and d, arrows). The corresponding post-contrast T1-weighted image and region-of-interest in this slice are shown (e). The percentage signal recovery of the mass is 1.50 (f). nADCmean = normalized mean apparent diffusion coefficient.

**Fig 2:**
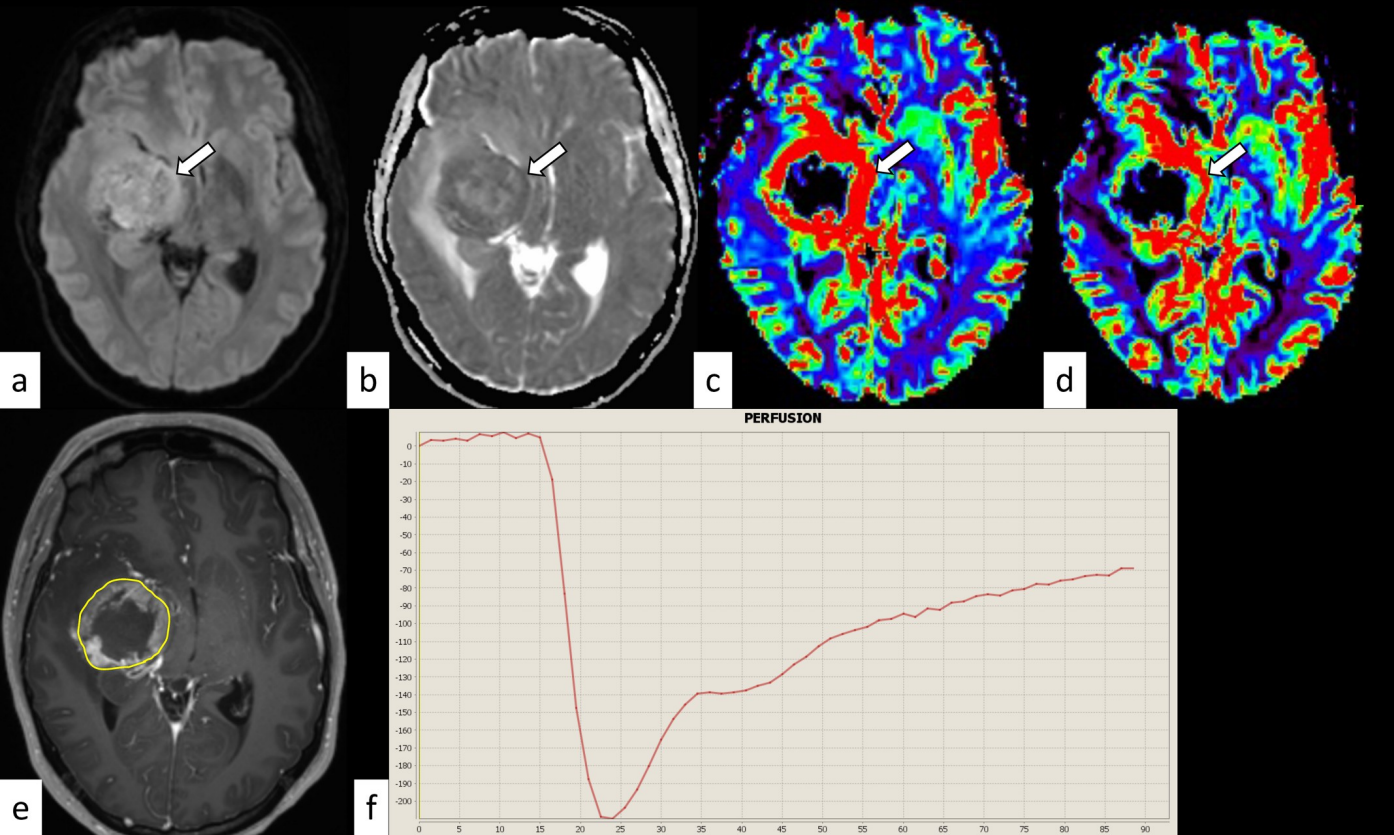
H3.3 variant diffuse midline glioma, H3K27-altered in a 28-year-old woman. The mass shows hyperintensity on diffusion-weighted imaging (a, arrow) and a decreased apparent diffusion coefficient (nADCmean is 1.18) (b, arrow). The normalized relative cerebral blood volume and flow are 2.93 and 2.10, respectively (c and d, arrows). The corresponding post-contrast T1-weighted image and region-of-interest in this slice are shown (e). The percentage signal recovery of the mass is 0.63 (f). nADCmean = normalized mean apparent diffusion coefficient.

**Fig 3:**
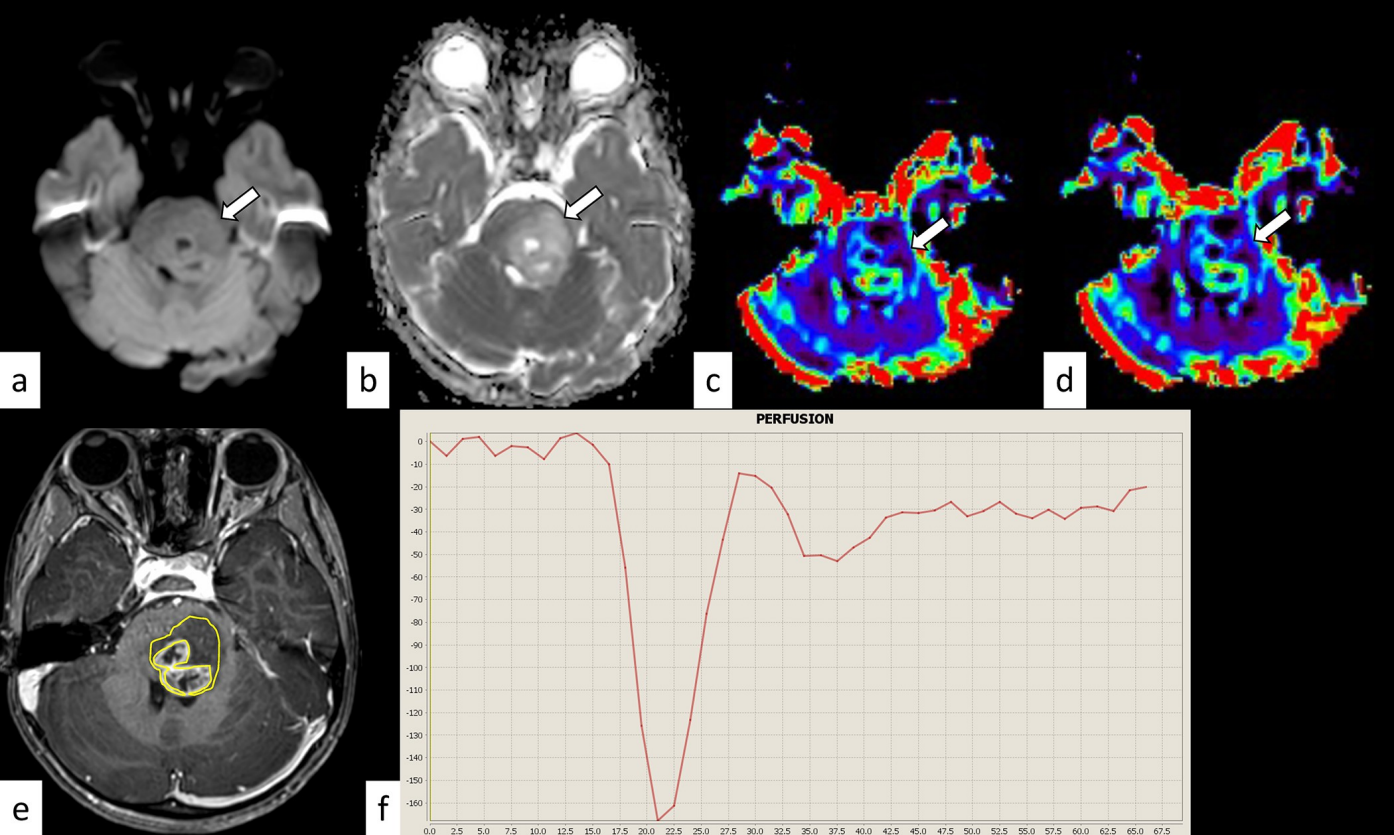
H3.1 variant diffuse midline glioma, H3K27-altered in a 4-year-old girl. The mass shows isointensity on diffusion-weighted imaging (a, arrow) and an increased apparent diffusion coefficient (nADCmean = 1.72) (b, arrow). The normalized relative cerebral blood volume and flow are 1.76 and 1.56, respectively (c and d, arrows). The corresponding post-contrast T1-weighted image and region-of-interest in this slice are shown (e). The percentage signal recovery of the mass is 0.78 (f). nADCmean = normalized mean apparent diffusion coefficient.

Sixteen patients with DMG (15 cases of H3.3 and one case of H3.1) and 14 patients with PA underwent DSC-MRI. All patients in this study underwent DWI. The nADCmean was significantly lower for DMG than that for PA (median: 1.48 vs. 1.96; *p* = 0.00075). The nADCmean tended to be lower for the H3.3 variant than that for the H3.1 variant (median: 1.46 vs. 1.80; *p* = 0.10), although the difference did not reach statistical significance. The nrCBV and nrCBF tended to be higher for DMG than that for PA (median nrCBV [DMG vs. PA]: 1.66 vs. 1.17, *p* = 0.058; and median nrCBF [DMG vs. PA]: 1.87 vs. 1.19, *p* = 0.028). PSR tended to be lower for DMG than that for PA (mean PSR: 0.97 vs. 1.23; *p* = 0.13). However, the differences were not statistically significant. Representative cases are shown in **Figs 1–3**.

## Discussion

In this study, we aimed to evaluate and compare the DSC parameters and ADC values between midline PA and DMG and between the H3.3 and H3.1 variants of DMG. We found that, compared with midline PA, DMG had a significantly lower nADCmean and tended to have a higher nrCBV and nrCBF and lower PSR. The nADCmean tended to be lower for the H3.3 variant than that for the H3.1 variant. To the best of our knowledge, this study is the first to compare the DSC parameters and ADC between midline PA and DMG and between the H3.3 and H3.1 variants of DMG.

DMG is a primary brain tumor that accounts for 50% of all pediatric high-grade gliomas, and it has a median overall survival of 9–11 months after diagnosis [18, 19]. Its prognosis is considerably more unfavorable than that of PA, which also often develops in the midline regions of the brain in children and is potentially curable via surgical resection. In contrast, recent advances in molecular genetics have led to the development of several potentially promising drugs for the treatment of DMG, such as targeted therapy for the platelet-derived growth factor receptor [20], ID1 [21], and dopamine receptor D2/3 [22] and GD2-directed chimeric antigen receptor T cell therapy [13]. Concurrent with the development of such medications, recognizing the differences and similarities in the imaging characteristics of DMG and its mimickers is crucial. In the present study, we found that the nADCmean was significantly lower for DMG than that for midline PA, possibly due to the different histopathological characteristics of the tumors. The cellularity of PA is low to moderate with varying degrees of myxoid background materials [23]. However, considerable overlap existed between the nADCmean for PA (range, 1.45–3.5) and DMG (range, 1.11–2.76). This finding may be because of the variations in the histopathology of these tumors. Some PAs tend to have a low myxoid stroma and a low ADC, whereas DMGs have a high myxoid stroma and a high ADC [24]. The H3.3 variant tended to have a lower nADCmean than the H3.1 variant in the present study. This finding may be related to the difference in the survival status between the variants. A significantly longer overall survival has been reported for patients with the H3.1 variant than for those with the H3.3 variant [5, 19]. Moreover, Aboian et al. [24] reported a significantly lower ADC in patients with DMG who survived <1 year after diagnosis than that in patients who survived >1 year. However, the details regarding the H3 variant in their study are unknown. Owing to the rarity of the H3.1 variant, only five H3.1 cases could be included in the present study. Including more H3.1 cases may clarify the reason behind the difference observed in the nADCmean between the two variants. The nrCBV and nrCBF tended to be higher, and PSR tended to be lower for DMG than those for midline PA; however, the differences did not reach statistical significance. Previous studies [25–27] have shown significantly lower nrCBV and nrCBF for DMG than those for H3-wildtype high-grade gliomas. In addition, lesser contrast enhancement has been reported in DMG than that in the H3-wildtype glioblastoma [28, 29]. Molecularly, it has been suggested that tumor-associated blood vessels in DMGs mainly consist of existing mature vasculature, not newly created vessels [30]. The unique molecular and radiological features of DMG resulting in poor contrast enhancement for CNS WHO grade 4 glioma may partially account for the overlap of the DSC parameters between DMG and PA in the present study.

This study had some limitations. First, it was a single-center retrospective study, and not all patients underwent DSC-MRI. These factors may have led to insufficient analysis of the DSC parameters. Second, owing to the rarity of the disease, only five patients with the H3.1 variant of DMG were included, and only one patient underwent DSC-MRI. Future studies with a greater number of H3.1 cases are necessary to further clarify the characteristics of DSC and ADC in this variant. Third, the MRI protocols were not homogeneous owing to the differences in the MRI systems. However, we mitigated the risk of heterogeneity for MRI parameters with normalization.

The nADCmean was significantly lower, the nrCBV and nrCBF tended to be higher, and PSR tended to be lower in DMG than those in midline PA. The H3.3 variant DMG tended to have a lower nADCmean than the H3.1 variant. Recognizing these differences and similarities in the DSC parameters and ADCs between DMG and midline PA and between the H3.3 and H3.1 variants is important for the appropriate management of these tumors.
